# Caregiver’s quality of life and its positive impact on symptomatology and quality of life of patients with schizophrenia

**DOI:** 10.1186/s12955-017-0652-6

**Published:** 2017-04-19

**Authors:** Alejandra Caqueo-Urízar, Marine Alessandrini, Alfonso Urzúa, Xavier Zendjidjian, Laurent Boyer, David R. Williams

**Affiliations:** 1 Escuela de Psicología y Filosofía, Universidad de Tarapacá, Avenida 18 de Septiembre 2222, Arica, Chile; 2 Department of Social and Behavioral Sciences, Harvard T. H. Chan School of Public Health, 677 Huntington Avenue, Boston, MA 02115-6018 USA; 3 Assistance publique des hôpitaux de Marseille, Hôpital de la Conception, pôle psychiatrie centre, 13005 Marseille, France; 4 Chronic Diseases and Quality of Life – Research Unit, Aix-Marseille University, EA 3279 – Public Health, 13005 Marseille, France; 5 Universidad Católica del Norte, Avda. Angamos 0610, Antofagasta, Chile; 6 Department of African and African American Studies, Harvard University, Cambridge, MA USA

**Keywords:** Schizophrenia, Caregiver, Quality of life, Structural equation modeling

## Abstract

**Background:**

Although the quality of life (QoL) experienced by patients with schizophrenia has been recognized, few studies have assessed the relationship between the caregivers’ QoL and patients’ QoL.

**Methods:**

The study included 253 stabilized outpatients with schizophrenia and their caregivers from 3 Mental Health Services in Bolivia (*N* = 83), Chile (*N* = 85) and Peru (*N* = 85). Caregivers’ and patients’ QoL were respectively assessed using two specific QoL questionnaires (S-CGQoL and S-QoL 18). We collected socio-demographic information and clinical data. Multiple linear regressions were performed to determine which variables were associated with patient’s QoL. We tested the following hypothesis using structural equation modeling (SEM): caregivers’ QoL may have an indirect effect on patients’ QoL mediated by their influence of the severity of psychotic symptoms.

**Results:**

In the multivariate analysis, the caregivers’ QoL was not significantly associated with the patients’ QoL, except for one QoL dimension about relationship with family (Beta = 0.23). Among patients’ characteristics, being a woman and Aymara, having lower educational level, unemployment and severity of symptoms was significantly associated to a lower QoL. The SEM revealed a moderate significant association between caregivers’ QoL and psychotic symptoms severity (path coefficient = −0.32) and a significant association between psychotic symptoms severity and patients QoL (path coefficient = −0.40). The indirect effect of caregivers’ QoL on patients’ QoL was significant (mediated effect coefficient = 0.13).

**Conclusion:**

Improvement of caregiver’s QoL may have a direct impact on the psychotic symptoms of patients and indirectly on patient’s QoL, confirming the need for ongoing family interventions in these regions.

## Background

Quality of life (QoL) measurements have become an important way to evaluate the treatments and care provided to patients with schizophrenia [1, 2]. QoL provides important information concerning the emotional and social experience of individuals which is not available for traditional assessments [3, 4]. In recent studies, QoL has been reported to be an independent predictor for long-term symptomatic remission, functional recovery and disability [5, 6]. Knowledge of the factors that are determinants of QoL in patients with schizophrenia may assist clinicians in choosing the most appropriate and effective interventions. However, the determinants of QoL remain poorly understood in this population. Recent works have argued for the need for a better understanding of the variables that contribute to QoL [7].

Over the last decades, numerous studies have investigated the value of psychotic symptoms, depression, neurocognition and functioning as predictors of QoL [7–10]. These studies have unquestionably advanced our knowledge of the determinants of QoL but they did not examine the influence of the caregivers on the QoL of patients despite their central role in caring for patients with schizophrenia. The caregivers are the individuals, generally a member of the patient’s family, who assume the role of care historically performed by psychiatric hospitals by providing informal care to patients since the large-scale deinstitutionalization of psychiatric patients [11, 12]. The caregiver spends most of his/her time caring for the patient, supplying support, checking medication and other aspects of the patient’s daily life.

Although they play a major role in providing care, the lack of interventions for counseling, training or supporting them has been highlighted [13].

Importantly, caregivers’ negative experiences may affect their ability to care for the patients and considering caregivers’ QoL may be of importance both for the caregivers themselves and indirectly for patients’ health. To our knowledge, no study has explored the association between the caregiver’s QoL and the symptomatology and quality of life of patients with schizophrenia. Such information is important to developing better services for caregivers.

The aim of this study was thus to determine whether caregivers’ QoL is a determinant of patients’ QoL from three countries in Latin America, while considering other important determinants such as socio-demographic and clinical characteristics. We hypothesize that caregivers’ QoL may have an indirect effect on patients’ QoL mediated by their influence on the severity of psychotic symptoms. We used structural equation modeling (SEM), which is a useful statistical procedure, to test a theory involving non-straightforward relationships and is therefore well suited to the management of cross-sectional data for inferential purposes [14].

## Method

### Study participants

This cross-sectional study evaluated data collected through a survey of patients and their primary caregivers conducted from May 2012 to February 2013 through the Public Mental Health Services program in three Latin American cities: La Paz, Bolivia (32.8%); Arica, Chile (33.6%), and Tacna, Peru (33.6%). Patients were invited to participate as they came to their monthly follow-up visits, usually accompanied by their key caregiver, defined as the person who fulfilled the primary caring role and spent more time than anyone else with the patient in the task of caring. Most of the people agreed to participate.

The inclusion criteria for patients were the following: being over 18 years of age and having a diagnosis of schizophrenia according to the criteria of the International Classification of Diseases, 10th version [15]. The inclusion criteria for the caregivers were the following: being identified by the individual with schizophrenia as the main caregiver and being 18 years of age or older. We applied a small set of exclusion criteria to the patient (being in a state of psychotic crisis or having a sensory or cognitive type of disorder that prevented being evaluated) and caregiver (presence of organic symptomatology; having a psychoactive substance abuse disorder; having a sensory or cognitive type of disorder that prevented being interviewed) groups to ensure the ability to participate fully in the interviews. These criteria were applied by the treating psychiatrist.

The sample included both Aymara and non-Aymara patients and caregivers. Aymara patients and caregivers were identified by Aymara surnames as established by legislation regarding indigenous peoples in the three countries, or Aymara self-identification. Both the Aymara and non-Aymara patients live in the same urban areas, are served by the same mental health centers, and have roughly comparable socio-demographic characteristics.

### Measures

#### Schizophrenia quality of life questionnaire (SQoL18) [16]

The SQoL18 is a self-administered QoL questionnaire designed for people with schizophrenia and has been used extensively in Europe [6, 17, 18] and Latin America [19]. It is a multidimensional instrument that exclusively assesses the patient’s view of his or her current QoL. It comprises 18 items describing 8 dimensions: psychological well-being (PsW), self-esteem (SE), family relationships (RFa), relationships with friends (RFr), resilience (RE), physical well-being (PhW), autonomy (AU), and sentimental life (SL), as well as a total score (index). Dimension and index scores range from 0, indicating the lowest QoL, to 100, the highest QoL.

#### Positive and negative syndrome scale for schizophrenia (PANSS) [20]

This is a 30-item, 7-point (1–7) rating scale that is specifically developed to assess psychotic symptoms in individuals with schizophrenia. This instrument is clinician-rated. For the purposes of this study, we considered the five subscales of the PANSS: positive, negative, excitation, anxiety/depression and cognitive subscales [21]. The PANSS has been translated and validated in Spain by Peralta and Cuesta (1994) [22] and also Fresán et al., (2005) [23] examined the psychometric properties of this instrument in Mexico. Positive and Negative Syndrome scale for Schizophrenia (PANSS) severity score are: PANSS total score of 58 = “Mildly ill”; PANSS total score of 75 = “Moderately ill”; PANSS total score of 95 = “Markedly ill” and PANSS total score of 116 = “Severely ill”.

#### Schizophrenia caregiver quality of life questionnaire (S-CGQoL) [24]

This instrument has 25 items and assesses QoL on seven dimensions. This questionnaire took about 5 min to administer and has satisfactory psychometric properties. Its structure explains 74.4% of the total variance, while its internal consistency, Cronbach’s coefficient alpha, ranges from 0.79 to 0.92 on the various dimensions: Psychological and Physical Well-being; Psychological Burden and Daily Life; Relationships with Spouse; Relationships with Psychiatric Team; Relationships with Family; Relationships with Friends; Material Burden and Total Index. Each dimension and the total index score range from 0, indicating the lowest QoL, to 100, the highest QoL.

#### Demographic and clinical data

For patients, we collected age, gender, ethnicity (Aymara and non-Aymara), marital status (with a partner/without a partner), educational level (≥12 years or < 12), employment status (with employment/without employment), family income (measure of the total salary per month for all members of the family, expressed in US dollars), duration of the disorder in years, number of hospitalizations (since the last 3 years before present hospitalization) and type of treatment (whether the patient received only pharmacological treatment from the mental health services or integrated treatment, meaning pharmacological plus psychotherapy, family psychoeducation, day care hospital). For caregivers, we collected age, gender, relationship with the patient (mother/other), marital status (with a partner/without a partner) and educational level (≥12 years or < 12).

### Procedures

The study was approved by the Ethics Committee of the University of Tarapacá and the National Health Service of Chile. We also obtained the authorization of the Mental Health Services in Peru and Bolivia. Two psychologists, who were part of the research team and supervised by the principal researcher, conducted the evaluations of family members and caregivers under the auspices of the mental health services of each country. The length of time of the evaluation of the caregivers was between 15 and 20 min; the evaluation of the patients was more extensive, between 30 and 40 min.

Before the start of the survey, informed consent was requested and received from the relative and the patient. The objectives of the study were explained as well as the voluntary nature of participation. No compensation was offered for participating in the study.

### Statistical analysis

Data were expressed as proportions or as the means, standard deviations, medians, and interquartile ranges (IQR). Associations between patients’ QoL scores (SQoL18) and the continuous variables (caregiver: S-CGQoL and patient: age, monthly family income, duration of disorder, number of hospitalizations, PANSS total score and its five dimensions, PANSS negative, positive, anxiety/depression and cognition) were analysed using Spearman’s correlation tests. Means-based comparisons of the SQoL18 index and dimensions between various sub-groups (gender, ethnicity, marital status, educational level, employment status and type of treatment) were calculated using Student t-tests.

Multiple linear regression analyses were then performed to identify variables potentially associated with patients’ QoL levels. The SQoL18 index and each of its dimensions were considered as separate dependent variables. The variables relevant to the models were selected from the bivariate SQoL18 index analysis based on a threshold *p*-value ≤0.20. Patients’ gender was included in the models owing to its clinical and socio-demographic interest. The final models incorporated the standardised ß coefficients, which represent a change in the standard deviation of the dependent variable (SQoL18) resulting from a change of one standard deviation in the various independent variables. The independent variables with the higher standardised beta coefficients are those with a greater relative effect on patients’ QoL.

**Fig. 1:**
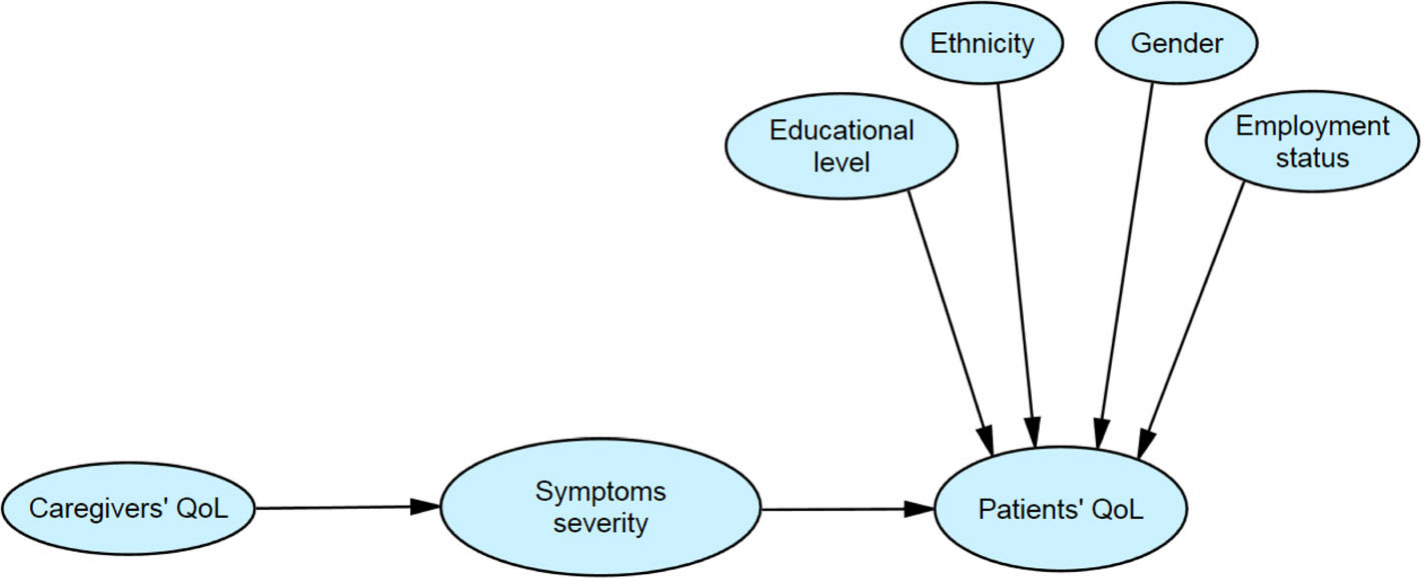
Hypothetical sequential process. QoL: Quality of Life

Finally, we assessed hypothetical relationships among the significant variables of the multivariate analyses to apply structural equation modeling (SEM). We hypothesized that caregivers’ QoL had an indirect effect on patients’ QoL mediated by their influence of the severity of psychotic symptoms. This model was adjusted for the significant covariates of the multivariate analyses. The model tested is presented in Fig. 1. Our model was based on three latent variables, caregivers QoL, psychotic symptoms severity and patients’ QoL. Socio-demographic characteristics (i.e. gender, ethnicity, educational level and employment status) are observed variables. We evaluated the model fit using the chi-squared statistic with the normed chi-square (χ^2^/df), the Root Mean Square Error of Approximation (RMSEA), the Comparative Fit Index (CFI) and the Standardized Root Mean Square Residuals (SRMR). The significance of the path coefficient was assessed using the standard errors and the t-values for each coefficient. In addition to the statistical significance of the path coefficients, the strength of the relationship plays a role in determining whether the relationships are weak (<0.2), moderate (0.2–0.5) or strong (>0.5) [25].

All the tests were two-sided. Statistical significance was defined as *p* < 0.05. The statistical analyses were performed using the SPSS version 20.0 software package (SPSS Inc., Chicago, IL, USA) MPLUS for the SEM analyses.

## Results

### Sample characteristics

Sample characteristics are presented in Table 1. Two hundred and fifty three patients with schizophrenia and their primary caregivers participated in our study. The mean age of patients was 35.6 years (±12.5 years) and 66.4% (*n* = 164) were male. The mean duration of illness was 11 years (IQR [5; 22]), and they had a moderate severity of psychotic symptoms with a total PANSS score of 71.3 (±28.2).

**Table 1:** Sample characteristics (*N* = 253)

Patients		Mean ± SD, median [IQR] or n (%)^a^
Age in years		35.6 ± 12.5
Gender	Women	83 (33.6)
	Men	164 (66.4)
Ethnicity	Non-Aymara	136 (53.8)
	Aymara	117 (46.2)
Marital Status	Without a partner	237 (93.7)
	With a partner	16 (6.3)
Educational level	≥12 years	40 (15.8)
	<12 years	213 (84.2)
Employment status	With employment	78 (31.2)
	Without employment	172 (68.8)
Monthly family income (US dollars)		331.3 [144.9; 517.9]
Duration of disorder in years		11 [5; 22]
Number of hospitalizations (since the last 3 years before present hospitalization)		1 [2; 0]
Type of mental health treatment	Integrated	31 (12.3)
	Only pharmacological	222 (87.7)
Symptoms severity	PANSS total score	71.3 ± 28.2
	*PANSS negative*	18.6 ± 8.4
	*PANSS positive*	8.3 ± 4.6
	*PANSS excitation*	11.5 ± 5.9
	*PANSS anxiety*/*depression*	6.4 ± 3.7
	*PANSS cognition*	7.2 ± 4.0
Quality of life	S-QoL 18 index	54.3 ± 14.4
Caregivers		
Age in years		54.7 ± 14.4
Gender		
	Women	170 (67.7)
	Men	81 (32.3)
Relationships with patient		
	Others	138 (54.5)
	Mothers	115 (45.5)
Marital Status	Without a partner	132 (52.2)
	With a partner	121 (47.8)
Educational level		
	≥12 years	66 (26.1)
	<12 years	187 (73.9)
Quality of life	S-CGQoL index	47.8 ± 15.7

^a^Mean ± SD: mean ± standard deviation; median [IQR]: median [Inter Quartile Range]; n (%): effective (percentage)

*PANSS* positive and negative syndrome scale for schizophrenia, total score and dimensions

*S*-*QoL*18 schizophrenia quality of life questionnaire

*S*-*CGQoL* schizophrenia caregiver quality of life questionnaire

### Factors associated to patient’s QoL

The results of the univariate and multivariate analyses are provided in Table 2. In the multivariate analysis, the caregivers’ QoL was not significantly associated with the patients’ QoL, except for the RFa dimension (r = 0.23). Among patients’ characteristics, being a woman and Aymara, having lower educational level, unemployment and higher severity of symptoms was significantly associated with a lower patients’ QoL.

**Table 2:** Factors associated with S-QoL18 index and dimensions

Univariate analysis				Multivariate analysis								
		S-QoL18 index	*p*	S-QoL18 index	PsW	SE	RFa	RFr	RE	PhW	AU	SL
		M ± SD^a^ or R^b^		ß^c^	ß	ß	ß	ß	ß	ß	ß	ß
Caregivers												
Quality of life	S-CGQoL	0.14	0.032	0.05	0.09	−0.12	0.23**	0.10	−0.15	−0.10	−0.09	0.11
Patients												
Age in years		−0.09	0.142	−0.12	−0.17	0.09	−0.14	−0.12	−0.16	0.02	0.14	−0.16
Gender	Women	52.7 ± 15.7	0.208	−0.20*	0.11	−0.07	−0.45**	−0.10	−0.17	−0.10	−0.19	−0.03
	Men (Ref)	55.1 ± 13.8										
Ethnicity	Non-Aymara	58.7 ± 17.2	0.035	0.19*	0.13	0.00	0.00	0.28**	0.04	0.00	0.00	0.22*
	Aymara (Ref)	52.3 ± 14.2										
Marital status	Without a partner	54.2 ± 14.6	0.794	-	-	-	-	-	-	-	-	-
	With a partner (Ref)	53.2 ± 13.4										
Educational level	≥12 years	62.5 ± 11.5	<0.001	0.14	0.31**	0.05	−0.04	0.05	0.05	0.04	0.06	0.12
	<12 years (Ref)	52.7 ± 14.4										
Employment status	With employment	59.2 ± 13.4	<0.001	0.15	0.082	0.15	0.02	0.12	0.18*	0.06	0.09	0.01
	Without employment (Ref)	52.1 ± 14.5										
Monthly family income (US Dollars)		0.08	0.246	-	-	-	-	-	-	-	-	-
Duration of disorder in years		−0.05	0.399	-	-	-	-	-	-	-	-	-
Number of hospitalizations		−0.09	0.168	−0.03	0.05	0.03	0.11	−0.15	−0.04	−0.01	0.11	−0.11
Type of treatment	Integrated	58.6 ± 11.8	0.076	0.06	0.11	0.13	−0.07	−0.01	0.12	0.15	−0.01	−0.07
	Only pharmacological (Ref)	53.7 ± 14.7										
Symptoms severity - PANSS total score		−0.37	<0.001	−0.23*	−0.27**	−0.15	−0.10	−0.11	−0.01	−0.12	−0.22*	−0.10
*PANSS negative*		−0.35	<0.001	-	-	-	-	-	-	-	-	-
*PANSS positive*		−0.26	<0.001	-	-	-	-	-	-	-	-	-
*PANSS excitation*		−0.24	<0.001	-	-	-	-	-	-	-	-	-
*PANSS anxiety*/*depression*		−0.17	0.006	-	-	-	-	-	-	-	-	-
*PANSS cognition*		−0.30	<0.001	-	-	-	-	-	-	-	-	-

^a^M ± SD: mean ± standard deviation; ^b^R: Spearman’s correlation coefficient; ^c^ß: standardised beta coefficient (ß represents the change of the standard deviation in QoL score resulting from a change of one standard deviation in the independent variable); *p ≤ 0.05; **p ≤ 0.01

*S*-*QoL*18 schizophrenia quality of life questionnaire, *PsW* psychological well-being, *SE* self-esteem, *RFa* family relationships, *RFr* relationships with friends, *RE* resilience, *PhW* physical well-being, *AU* autonomy, *SL* sentimental life

Number of hospitalizations: since the last 3 years before present hospitalization

*PANSS* positive and negative syndrome scale for schizophrenia, total score and dimensions

*S*-*CGQoL* schizophrenia caregiver quality of life questionnaire

### Structural equation model

**Fig. 2:**
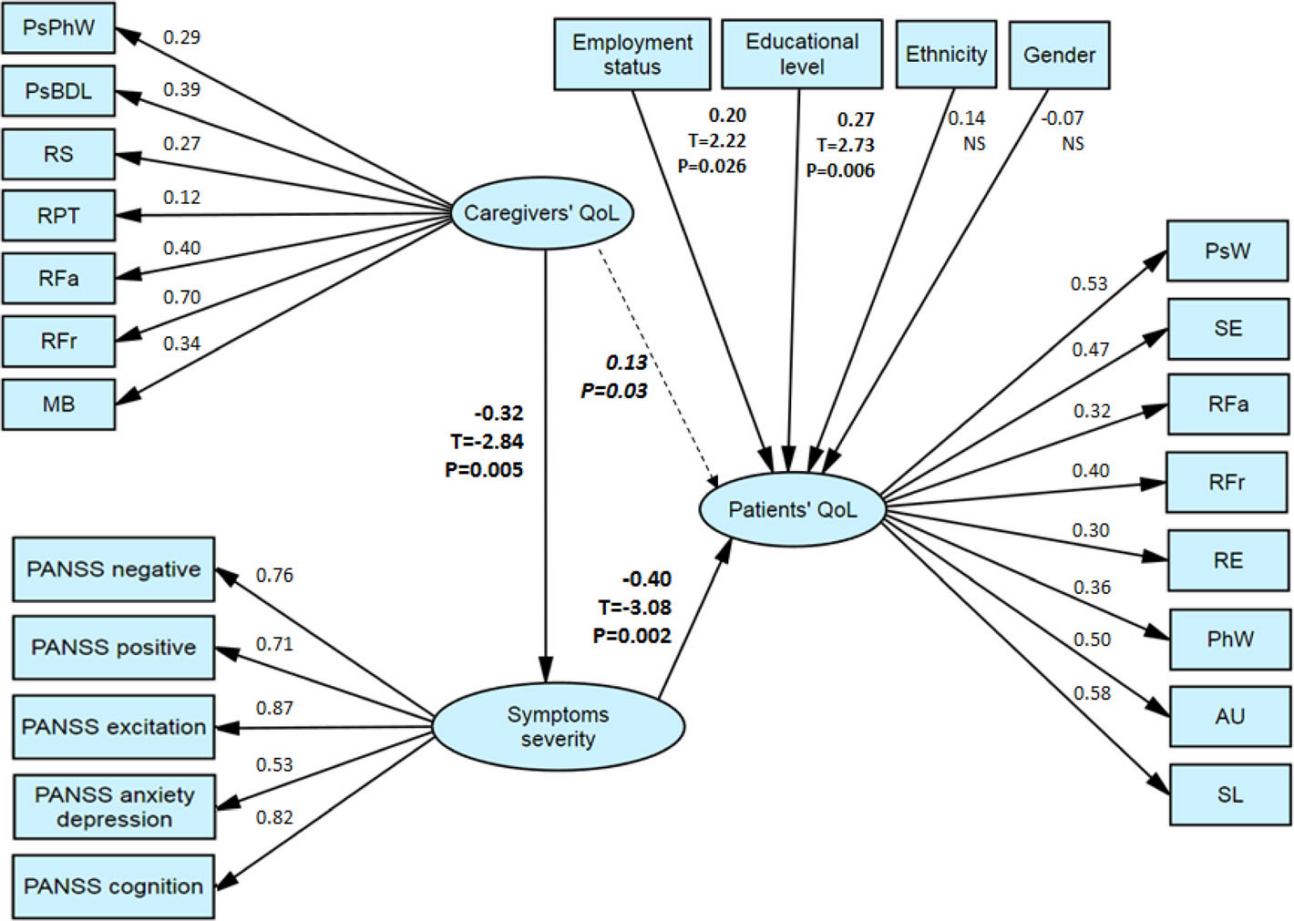
Structural equation model (SEM) with fitted coefficients. SEM goodness of fit: chi square = 384.39 DF = 231 normed chi square = 1.66 CFI = 0.88 RMSEA = 0.05 and SRMR = 0.05. Patients’ QoL, symptoms severity and patients’ QoL are latent variables (with arrows pointing to their respective indicators). The patients’ QoL is composed of the 8 dimensions of the S-QoL 18: Schizophrenia Quality of Life 18 items; PsW: Psychological Well-being dimension of the S-QoL 18; SE: Self-Esteem; RFa: Family Relationships; RFr: Relationships with Friends; RE: Resilience; PhW: Physical Well-being; AU: Autonomy; and SL: Sentimental Life. The psychotic symptoms severity is composed of the 5 factors of the PANSS (Positive and Negative Syndrome Scale): negative, positive, excitation, anxiety/depression and cognitive factors. The caregivers’ QoL is composed of the 7 dimensions of the S-CGQoL (Schizophrenia Caregiver Quality of Life questionnaire): PsPhW (Psychological and Physical Well-being); PsBDL (Psychological Burden and Daily Life); RS (Relationships with Spouse); RPT: Relationships with Psychiatric Team; RFa: Relationships with Family; RFr: Relationships with Friends; MB: Material Burden. Educational level, ethnicity, gender and employment status are independent factors (observed variables)

The SEM is presented in Fig. 2 confirming our hypothesis. The SEM showed good fit with χ^2^/df = 1.66, root mean square error of approximation RMSEA = 0.05, comparative fit index CFI = 0.88 and standardized root mean square residuals SRMR = 0.05. The SEM revealed a moderate significant association between caregivers’ QoL and psychotic symptoms severity (path coefficient = −0.32) and a significant association between psychotic symptoms severity and patients QoL (path coefficient = −0.40). The indirect effect of caregivers’ QoL on patients’ QoL was significant (mediated effect coefficient = 0.13). The psychotic symptoms severity was not equally associated with caregivers’ and patients’ QoL. The PANSS excitation was the most important factor loaded (0.87) while the PANSS depression was the least important (0.53).

## Discussion

First, at the patient level, as in previous studies, socio-cultural and economic factors were associated with patient’s QoL in this sample: being female, having a lower educational level, being unemployed and belonging to an ethnic minority (i.e., Aymara) was associated with lower reports of QoL [26–28]. These results are also consistent with previous studies indicating that a higher level of education facilitates employment, thus improving patients’ level of income and QoL [29–32]. An explanation of the results for QoL associated with ethnic minority status can relate to the fact that this population, with 2 million people, have participated in a massive migration from the Andes Mountains to large cities searching for a brighter future; it is likely that in this process they encountered discrimination based on their Andes phenotype [33–38]. Prior researches from multiple societies revealed that ethnic minorities are exposed to discrimination and that these stressful experiences adversely affect physical and mental health and therefore their QoL [39–44]. Symptoms severity was also associated with patient’s QoL. Several studies including meta-analyses have reported this association although the percentage of variance explained by symptoms remains moderate in comparison to socioeconomic factors [45–50]. The analyses from this sample show that the control of symptoms remains an important need for patients with schizophrenia living in Latin America. This finding may be not surprising in Latin America where the WHO Report on mental health systems identified the absence of universal social security coverage for mental disorders, the lack of capacity for some patients to purchase antipsychotic medications and the limited role of primary care in the mental health area [51].

Second, in relation to caregiver level, it is necessary to mention that the change in the way patients with schizophrenia are treated (de-institutionalization) has resulted in a dramatic shift in the burden of caregiving from health care professionals to family members [52]. The chronic illness of a family member can be a source of stress for the caregiver who can appraise this stressor as one with a high level of demands in relation to caregiving. The stress-appraisal-coping model suggests that during this process, the family member takes into consideration the nature of the stressor and the resources to cope [53, 54]. The impact of the caregiver’s role depends on the characteristics of the patient, the family member, their relationship and the environment [55].

The results of this study show that there is a significant association between caregivers’ QoL and patients’ QoL mediated by psychotic symptoms severity. Previous studies have shown that patients’ clinical variables are those that most significantly affect caregivers’ QoL, such as: duration of the disorder, patient’s disruptive behavior, lower social functioning, higher level of disability, positive and negatives symptoms and general psychopathology [4, 56–62]. For the first time, this study shows that caregivers’ QoL may have a positive association with psychotic symptoms severity and patients’ QoL, confirming the central role of caregivers in the treatment of patients.

While the majority of studies in this substantive area have been conducted in European and North American countries, there have been few prior research studies in Latin America. Thus, these findings are a contribution to the existing evidence on the importance of considering the caregiver as an ally and indispensable agent in the treatment of the patient. However, especially in low and middle-income countries, there is a lack of systematic intervention to enhance the capacity and provide resources to these families. Once the psychoeducation program is completed, studies show that effective treatment for patients with schizophrenia needs further monitoring and involvement of health professionals because both family and patients will face long-term challenges, needing support and strategies to cope with these difficulties, and hence the service to the patient and family should be permanent [63]. However, one of the biggest barriers to achieve this type of family treatment is of an economic nature, both, at the *macro* level (such as the lack of professionals in public health services), as well as *micro* level, where even when there are family interventions as a part of the mental health services, caregivers may lack resources to take advantage of them, such as not having enough money for transportation to these services [64].

Family interventions can improve caregivers’ QoL, however, they may also benefit from programs to reduce the level and chronicity of poverty [65], which influence directly patient’s QoL as previously reported in our findings. Indeed, growing international evidence shows that mental illness and poverty interact in a negative cycle: “poverty breeds ill-health, and ill-health keeps poor people poor” [66, 67]. Poverty worsens the health of patients with schizophrenia, and increases the burden of caregivers, which in turn may affect the caregivers’ health and their ability to care for the patients, so improvement of caregivers’ QoL should be a multidisciplinary, comprehensive effort [68–70].

This study had some limitations that should be considered. First, the sample is not representative of the entire Latin American population of caregivers and patients with schizophrenia. Larger studies with more diverse groups of patients and caregivers are needed to confirm our findings. Second, our study used only one type of QoL instrument for each participant. It would be interesting to determine whether our findings would be replicated with QoL instruments that utilize other conceptual models and dimensional constructs. Third, this study only provided information about the main clinical characteristics of our sample, and did not report further details concerning clinical stability and prescribed medication, for example. Fourth, the data are cross-sectional and even though we use SEM, we are unable to make causal claims. Fifth, it was not statistically possible to test simultaneously in our SEM model the existence of the indirect effect of caregivers’ QoL on patients’ QoL and the existence of a bilateral association between caregivers’QoL and severity of symptoms. We thus tested another model with a bidirectional association between caregivers’ QoL and severity of symptoms (but without testing the indirect effect of caregivers’ QoL on patients’ QoL). This model confirmed that a bidirectional association is probable (path coefficient = −0.4, *p* < 0.001).

Nonetheless, the limitations of the study should be considered in the light of its strengths. This was the first study carried out in Latin America that considers the relationship between caregiver’ QoL and patient’ QoL. Moreover, in this multicentric, international study, we confirmed the key role of the caregiver in patient treatment and the necessity to consider his/her health and QoL in a comprehensive assessment of the needs of the patient.

## Conclusion

Improvement of caregivers’ QoL may have a direct impact on the psychotic symptoms of patients and indirectly on patients QoL. So caregivers’ QoL is a major concern and mental health professionals and policy makers should consider the establishment of routine and ongoing family interventions in Latin America.

## Abbreviations

AU: Autonomy
CFI: Comparative Fit Index
MB: Material Burden
PANSS: Positive and Negative Syndrome scale for Schizophrenia
PhW: Physical well-being
PsBDL: Psychological Burden and Daily Life
PsPhW: Psychological and Physical Well-being
PsW: Psychological well-being
QoL: Quality of life
RE: Resilience
RFa: Family relationships
Rfa: Relationships with Family
Rfr: Relationships with Friends
RFr: Relationships with friends
RMSEA: Root Mean Square Error of Approximation
RPT: Relationships with Psychiatric Team
RS: Relationships with Spouse
S-CGQoL: Schizophrenia Caregiver Quality of Life questionnaire
SE: Self-esteem
SEM: Structural equation modeling
SL: Sentimental life
S-QoL 18: Schizophrenia Quality of Life Questionnaire
SRMR: Standardized Root Mean Square Residuals
χ2/df: chi-squared statistic with the normed chi-square

## References

[1] Boyer L, Baumstarck K, Boucekine M, Blanc J, Lancon C, Auquier P (2013). Measuring quality of life in patients with schizophrenia: an overview. Expert Rev Pharmacoecon Outcomes Res.

[2] Awad AG, Voruganti LN, Heslegrave RJ (1997). Measuring quality of life in patients with schizophrenia. Pharmacoeconomics.

[3] Faget-Agius C, Boyer L, Jonathan W, Jean-Philippe R, Raphaelle R, Elisabeth S, Sylviane CG, Pascal A, Maxime G, Christophe L (2015). Neural substrate of quality of life in patients with schizophrenia: a magnetisation transfer imaging study. Sci Rep.

[4] Caqueo-Urízar A, Gutiérrez-Maldonado J, Ferrer-García M, Urzúa-Morales A, Fernández-Dávila P (2011). Typology of schizophrenic symptoms and quality of life in patients and their main caregivers of North of Chile. Int J Soc Psychiatry.

[5] Lambert M, Karow A, Leucht S, Schimmelmann BG, Naber D (2010). Remission in schizophrenia: validity, frequency, predictors, and patients’ perspective 5 years later. Dialogues Clin Neurosci.

[6] Boyer L, Millier A, Perthame E, Aballea S, Auquier P, Toumi M (2013). Quality of life is predictive of relapse in schizophrenia. BMC Psychiatry.

[7] Fervaha G, Agid O, Takeuchi H, Foussias G, Remington G (2013). Clinical determinants of life satisfaction in chronic schizophrenia: data from the CATIE study. Schizophr Res.

[8] Boyer L, Aghababian V, Richieri R, Loundou A, Padovani R, Simeoni MC, Auquier P, Lançon C (2012). Insight into illness, neurocognition and quality of life in schizophrenia Prog. Neuro-Psychopharmacol Biol Psychiatry.

[9] Chou C-Y, Ma M-C, Yang T-T (2014). Determinants of subjective health-related quality of life (HRQoL) for patients with schizophrenia. Schizophr Res.

[10] Meesters PD, Comijs HS, De Haan L, Smit JH, Eikelenboom P, Beekman ATF, Stek ML (2013). Subjective quality of life and its determinants in a catchment area based population of elderly schizophrenia patients. Schizophr Res.

[11] Zendjidjian XY, Boyer L (2014). Challenges in measuring outcomes for caregivers of people with mental health problems. Dialogues Clin Neurosci.

[12] Caqueo-Urízar A, Gutiérrez-Maldonado J, Miranda-Castillo C (2009). Quality of life in caregivers of patients with schizophrenia: a literature review. Health Qual Life Outcomes.

[13] Caqueo-Urízar A, Rus-Calafell M, Urzúa A, Escudero J, Gutiérrez-Maldonado J (2015). The role of family therapy in the management of schizophrenia: challenges and solutions. Neuropsychiatr Dis Treat.

[14] Falissard B, Falissard B (2005). Modèles structuraux (Structural models). Comprendre et utiliser les statistiques dans les sciences de la vie (Understanding and using statistics in life-related sciences).

[15] World Health Organisation (1992). ICD-10 Classifications of Mental and Behavioural Disorder: Clinical Descriptions and Diagnostic Guidelines.

[16] Boyer L, Simeoni MC, Loundou A, D’Amato T, Reine G, Lancon C, Auquier P (2010). The development of the S-QoL 18: a shortened quality of life questionnaire for patients with schizophrenia. Schizophr Res.

[17] Auquier P, Tinland A, Fortanier C, Loundou A, Baumstarck K, Lancon C, Boyer L (2013). Toward meeting the needs of homeless people with schizophrenia: the validity of quality of life measurement. PLoS One.

[18] Baumstarck K, Boucekine M, Klemina I, Reuter F, Aghababian V, Loundou A, Pelletier J, Auquier P (2013). What is the relevance of quality of life assessment for patients with attention impairment?. Health Qual Life Outcomes.

[19] Caqueo-Urízar A, Boyer L, Boucekine M, Auquier P (2014). Spanish cross-cultural adaptation and psychometric properties of the Schizophrenia Quality of Life short-version questionnaire (SQoL18) in 3 middle-income countries: Bolivia, Chile and Peru. Schizophr Res.

[20] Kay SR, Fiszbein A, Opler L (1987). The positive and negative syndrome scale (PANSS) for schizophrenia. Schizophr Bull.

[21] Lancon C, Auquier P, Nayt G, Reine G (2000). Stability of the five-factor structure of the Positive and Negative Syndrome Scale (PANSS). Schizophr Res.

[22] Peralta V, Cuesta MJ (1994). Validación de la Escala de los Síndromes Positivo y Negativo (PANSS) en una muestra de esquizofrénicos españoles. [Validation of the positive and negative syndrome scale (PANSS) in a sample of Spanish schizophrenic]. Actas Luso Esp Neurol Psiquiatr Cienc Afines.

[23] Fresán A, De la Fuente-Sandoval C, Loyzaga C, García-Anaya M, Meyenberg N, Nicolini H, Apiquian R (2005). A forced five-dimensional factor analysis and concurrent validity of the Positive and Negative Syndrome Scale in Mexican schizophrenic patients. Schizophr Res.

[24] Richieri R, Boyer L, Reine G, Loundou A, Auquier P, Lançon C, Simeoni M (2011). The Schizophrenia Caregiver Quality of Life questionnaire (S-CGQoL): development and validation of an instrument to measure quality of life of caregivers of individuals with schizophrenia. Schizophr Res.

[25] Cohen J (1982). Set correlation as a general multivariate data-analytic method. Multivariate Behav Res.

[26] Mercier C, Pe’ladeau N, Tempier R (1998). Age, gender and quality of life in schizophrenia. Community Ment Health J.

[27] Caron J, Lecomte Y, Emmanuel S, Renaud S (2005). Predictors of quality of life in schizophrenia. Community Ment Health J.

[28] Caqueo-Urízar A, Urzúa A, Boyer L, Williams DR (2016). Religion involvement and quality of life in patients with schizophrenia in Latin-America. Soc Psychiatry Psychiatr Epidemiol.

[29] Browne G, Courtney M (2005). Housing, social support and people with schizophrenia: a grounded theory study. Issues Ment Health Nurs.

[30] Marwaha S, Johnson S (2004). Schizophrenia and employment - a review. Soc Psychiatry Psychiatr Epidemiol.

[31] Ruggeri M, Nose M, Bonetto C, Cristofalo D, Lasalvia A, Salvi G, Benedetta S, Malchiodi F, Tansella M (2005). Changes and predictors of change in objective and subjective quality of life: multiwave follow-up study in community psychiatric practice. Br J Psychiatry.

[32] Schomerus G, Heider D, Angermeyer MC, Bebbington PE, Azorin JM, Brugha T, Toumi M (2007). European Schizophrenia Cohort. Residential area and social contacts in schizophrenia. Results from the European Schizophrenia Cohort (EuroSC). Soc Psychiatry Psychiatr Epidemiol.

[33] Köster G, Van den Berg H, Schiffers N (1992). Los Aymaras: Características demográficas de un grupo étnico indígena antiguo en los Andes centrales. The Aymara: Demographic characteristics of an ancient indigenous ethnic group in the Central Andes.

[34] Van Kessel J, Hidalgo J, Schiappacasse F, Niemeyer F, Aldunate C, Mege P, La cosmovisión Aymara (1996). Etnografía: Sociedades indígenas contemporáneas y su ideología. Ethnography: Contemporary indigenous societies and their ideology.

[35] Núñez R, Cornejo C (2012). Facing the sunrise: Cultural worldview underlying intrinsic-based encoding of absolute frames of reference in Aymara. Cognitive Sci.

[36] Gundermann H (2000). Las organizaciones étnicas y el discurso de la identidad en el norte de Chile, 1980–2000 [Ethnic organizations and the discourse of identity in the North of Chile, 1980–2000]. Estudios Atacameños.

[37] Zapata C (2007). Memoria e historia: El proyecto de una identidad colectiva entre los aymaras de Chile [Memory and history: the project of a collective identity among the Aymara of Chile]. Chungara.

[38] Kirberg A (2006). La salud del niño Aymara. Rev Chil Pediatr.

[39] Lewis TT, Cogburn CD, Williams DR (2015). Self-Reported Experiences of Discrimination and Health: Scientific Advances, Ongoing Controversies, and Emerging Issues. Annu Rev Clin Psychol.

[40] Thompson A, Carrasquillo O, Gameroff M, Weissman M (2010). Psychiatric Treatment Needs Among the Medically Underserved: A Study of Black and White Primary Care Patients Residing in a Racial Minority Neighborhood. Prim Care Companion J Clin Psychiatry.

[41] Kung W (2003). The illness, stigma, culture or inmigration? Burdens on Chinese – American caregivers of patients with schizophrenia. Fam Soc.

[42] Haas JS, Earle CC, Orav JE, Brawarsky P, Neville BA, Williams DR (2008). Racial segregation and disparities in cancer stage for seniors. J Gen Intern Med.

[43] Smith DB, Feng Z, Fennell ML, Zinn JS, Mor V (2007). Separate and unequal: racial segregation and disparities in quality across US nursing homes. Health Aff.

[44] Vicente B, Kohn B, Rioseco P, Saldivia S, Torres S (2005). Psychiatric Disorders among the Mapuche in Chile. Int J Soc Psychiatry.

[45] Meesters PD, Comijs HC, De Haan L, Smit JH, Eikelenboom P, Beekman AT, Stek ML (2011). Symptomatic remission and associated factors in a catchment area based population of older patients with schizophrenia. Schizophr Res.

[46] Karow A, Naber D, Lambert M, Moritz S (2012). EGOFORS Initiative. Remission as perceived by people with schizophrenia, family members and psychiatrists. Eur Psychiatry.

[47] Eack S, Newhill C (2007). Psychiatric Symptoms and Quality of Life in Schizophrenia: A Meta-Analysis. Schizophr Bull.

[48] Vatne S, Bjorkly S (2008). Empirical evidence for using subjective quality of life as an outcome variable in clinical studies A meta-analysis of correlates and predictors in persons with a major mental disorder living in the community. Clin Psychol Rev.

[49] Priebe S, Reininghaus U, McCabe R, Burns T, Eklund M, Hansson L, Junghan U, Kallert T, Van Nieuwenhuizen C, Ruggeri M, Slade M, Wang D (2011). Factors influencing subjective quality of life in patients with schizophrenia and other mental disorders: a pooled analysis. Schizophr Res.

[50] Galuppi A, Turola MC, Nanni MG, Mazzoni P, Grassi L (2010). Schizophrenia and quality of life: how important are symptoms and functioning?. Int J Ment Health Syst.

[51] WHO.World Health Organization. Report on the Assessment of Mental Health Systems in Latin America and the Caribbean using the World Health Organization Assessment Instrument for Mental Health Systems (WHO-AIMS). 2013 http://www.who.int/mental_health/evidence/WHO-AIMS/en/ Accessed 21 Jan 2014.

[52] Caqueo-Urízar A, Breslau J, Gilman S (2015). Beliefs about the causes of schizophrenia among Aymara and non-Aymara patients and their primary caregivers in the Central-Southern Andes. Int J Soc Psychiatry.

[53] Schene AH (1990). Objective and subjective dimensions of family burden. Towards an integrative framework for research. Soc Psychiatry Psychiatr Epidemiol.

[54] Kate N, Grover S, Kulhara P, Nehra R (2014). Relationship of quality of life with coping and burden in primary caregivers of patients with schizophrenia. Int J Soc Psychiatry.

[55] Zahid MA, Ohaeri JU (2010). Relationship of family caregiver burden with quality of care and psychopathology in a sample of Arab subjects with schizophrenia. BMC Psychiatry.

[56] Gutiérrez-Maldonado J, Caqueo-Urízar A, Ferrer-García M, Fernández-Dávila P (2012). Influencia de la percepción de apoyo y del funcionamiento social en la calidad de vida de pacientes con Esquizofrenia y sus cuidadores. Psicothema.

[57] Rabinowitz J, Berardo CG, Bugarski-Kirola D, Marder S (2013). Association of prominent positive and prominent negative symptoms and functional health, well-being, healthcare-related quality of life and family burden: a CATIE analysis. Schizophr Res.

[58] Nehra R, Chakrabarti S, Kulhara P, Sharma R (2006). Family burden and its correlates among caregivers of schizophrenia and bipolar affective disorder. J Mental Health Hum Behav.

[59] Creado DN, Parkar SR, Kamath RM (2006). A comparison of the level of functioning in chronic schizophrenia with coping and burden in caregivers. Indian J Psychiatry.

[60] Saldanha P, Pai N, Krishnamurthy K (2002). A study of family burden and family distress in schizophrenia. Indian J Soc Psychiatry.

[61] Thomas JK, Suresh Kumar PN, Verma AN, Sinha VK, Andrade C (2004). Psychosocial dysfunction and family burden in schizophrenia and obsessive compulsive disorder. Indian J Psychiatry.

[62] Kate N, Grover S, Kulhara P, Nehra R (2013). Relationship of caregiver burden with coping strategies, social support, psychological morbidity, and quality of life in the caregivers of schizophrenia. Asian J Psychiatr.

[63] Carrà G, Montomoli C, Clerici M, Cazzullo C (2007). Family interventions for schizophrenia in Italy: randomized controlled trial. Eur Arch Psychiatry Clin Neurosci.

[64] McFarlane W, Dixon L, Luckens E, Lucksted A (2003). Family Psychoeducation and Schizophrenia: A review of the literature. J Marital Fam Ther.

[65] Cunningham P, McKenzie K, Taylor EF (2006). The struggle to provide community-based care to low-income people with serious mental illnesses. Health Aff.

[66] Wagstaff A (2002). Poverty and health sector inequalities. Bull World Health Organ.

[67] Lund C, De Silva M, Plagerson S, Cooper S, Chisholm D, Das J, Knapp M, Patel V (2011). Poverty and mental disorders: breaking the cycle in low-income and middle-income countries. Lancet.

[68] Karanci AN (1995). Caregivers of Turkish schizophrenic patients: causal attributions, burdens and attitudes to help from the health professional. Soc Psychiatry Psychiatr Epidemiol.

[69] Butzlaff RL, Hooley JM (1998). Expressed emotion and psychiatric relapse: a meta-analysis. Arch Gen Psychiatry.

[70] Caqueo-Urízar A, Gutiérrez J (2006). Burden in families of patients with schizophrenia. Qual Life Res.

